# The metaphysics of evolution

**DOI:** 10.1098/rsfs.2016.0148

**Published:** 2017-08-18

**Authors:** John Dupré

**Affiliations:** Egenis, University of Exeter, Exeter, UK

**Keywords:** process ontology, evolution, species, lineage, individual

## Abstract

This paper briefly describes process metaphysics, and argues that it is better suited for describing life than the more standard thing, or substance, metaphysics. It then explores the implications of process metaphysics for conceptualizing evolution. After explaining what it is for an organism to be a process, the paper takes up the Hull/Ghiselin thesis of species as individuals and explores the conditions under which a species or lineage could constitute an individual process. It is argued that only sexual species satisfy these conditions, and that within sexual species the degree of organization varies. This, in turn, has important implications for species' evolvability. One important moral is that evolution will work differently in different biological domains.

## 1. Introduction: why metaphysics?

Metaphysics is the branch of philosophy that aspires to provide the most general description of reality. Metaphysics aims to say what exists, but at a more general and abstract level than that typical of practical science or, for that matter, everyday life. It may ask, for example, whether there is one kind of being, two (as Descartes believed), or many. It may ask about the relations between very broad categories of entities. Now almost all biologists believe that living beings are made of the same kind of material stuff as the non-living; once, however, it was common to suppose that investigating life involved investigating something in certain respects, at least, quite different from the vulgarly material. This, one might say, is an example of progress in metaphysics.

The last example also illustrates that, though they may sincerely deny it, scientists are almost inevitably committed to metaphysical opinions, and that these make a difference to their work. A biologist not committed to the materialist metaphysics mentioned in the last paragraph would not look for the fundamental understanding of life in the properties of specific kinds of matter. Metaphysics can be ignored but not escaped. In the words of theoretical biologist and philosopher Joseph Henry Woodger, ‘physiologists [who] suppose themselves to be above “metaphysics” […] are only a very little above it—being up to the neck in it’ [1, p. 246].

The metaphysics I am interested in is a naturalistic one, providing an *ontology*, an account of what exists, ultimately grounded in our best science. I have said that scientists cannot avoid metaphysical assumptions, but these need not be explicit. Philosophical analysis of scientific work may help to expose these assumptions. But philosophical reflection on scientific findings may also point to an ontology different in important respects from that originally assumed. One reason it may do so is that philosophical enquiry of this sort is free to range over all domains of scientific enquiry. One motivation for ontological enquiry is thus to explore the consistency of interpretation of scientific results across the sciences or their subfields.

The metaphysical question with which this paper is concerned is an ancient one, the debate whether the world is ultimately composed of things, perhaps eternal and immutable things as was proposed by the Greek atomists, or rather is everywhere in flux, as famously advocated by the Greek philosopher Heraclitus. For process philosophers, enduring things, rather than being the more or less unchanging furniture of the world, are ‘never more than patterns of stability in a sea of process’ [2].

The ontology of things, following the revival of atomism in the seventeenth century, has been the dominant metaphysics for most of the history of modern science. It is closely connected to a further position that underlies conceptions of scientific explanation, *mechanicism*. For mechanicism, the way to understand or explain a phenomenon is to identify the various constituent things that interact to generate the phenomenon. Arrangements of constituents with particular functions constitute *mechanisms*. Mechanicism sees living systems as composed of things arranged in a hierarchy of mechanisms. This is a strictly bottom-up perspective, related to, if generally distinguished from, the often criticized but still widely endorsed methodological approach of reductionism. Process ontologists generally reject both mechanicism and reductionism, for they notice that what maintains the patterns of stability in the sea of process is not only the behaviour of the entities that compose the pattern, but also the network of relations between the patterns and their surroundings [3].

The aim of this paper is to explore the relevance of process ontology to evolutionary theory. Of course, no one doubts that evolution itself is a process. Thing (or *substance*, as a particular, very influential version of the concept is often referred to in philosophical writing [4]) ontologists do not deny that there are processes; it is rather that they see processes as generally requiring things as their subjects, as what happens to things. For process ontology, evolution is also, of course, a process, but the organisms and the lineages that are the subject matter of evolution are themselves also processes. I shall try very briefly to justify these claims, and then examine some of the implications they involve for how we should think about evolution.

## 2. Process metaphysics

What is the difference between seeing some entity as a thing, on the one hand, or as a temporarily stable process, on the other? Consider two paradigm cases: a mountain and a storm. A mountain is naturally thought of as a fairly fixed part of the world's furnishings; if any major change befalls it we are entitled to wonder why. In fact, in the context of a more generalized process ontology, on the timescale of tectonics the mountain is very much a stage in a process: for process ontology, being a thing is always relative to a timescale. The mountain will, nonetheless, serve well enough as an intuitive paradigm for a static thing, deriving its stability from inertia. Philosophers have asked how it is even possible for a thing to change and yet remain the same thing through time, and they have generally answered by positing some core of essential properties that must remain fixed regardless of the extent of change.

A storm may also be a very stable element of the world. The Red Spot on Jupiter, for instance, has been observed for several centuries, albeit with gradual changes in shape and size. But unlike the mountain, the Red Spot does not persist because nothing happens to change it, but because a stable pattern is maintained by the very rapid winds that circulate round it. If this activity ceased the Red Spot would dissipate very quickly. Its persistence through time is understood not through unchanging essential properties but through the causal continuity of the processes that maintain the pattern. A process ontology for life starts with the idea that the Red Spot is a more useful paradigm for living systems than the mountain.

Two simple points should be sufficient to confirm the appropriateness of the dynamic, processual perspective for thinking of biological systems. Consider, as paradigm living systems, organisms. The first, decisive reason for taking organisms to be processes is that they are open systems, far from thermodynamic equilibrium. It is an elementary fact of physics that maintaining such a system will require constant interaction with, and intake of matter or energy from, the environment. Its persistence is actively maintained rather than just given. Stasis, for an organism, is death.

Second, organisms undergo developmental cycles. Consider for instance the typical life cycle of an insect, comprising the egg, larva, pupa, and adult. These stages have very different properties. It is unclear what properties could possibly support the claim that these developmental stages were all one and the same *thing*. What could be an essential property of such a thing? It is sometimes suggested that genome sequence might provide such an essential, continuing property for an organism. I have responded to this idea in detail in [5], but perhaps a sufficient response is to note the work that the cell has to do to sustain a sufficiently accurate sequence [6]: genome sequence is as much the consequence of organismic stability as it is its source. For a process, at any rate, no such constant property is required: persistence is something the organism achieves, not some property or properties that it continues to possess. A process is inherently extended in time, and whatever claims temporal parts of a process have to be parts of one and the same process derive rather from causal connections between these parts.

Let me now mention two reasons why the insistence that living systems are processes rather than things matters. The first is that it motivates a significant shift in emphasis with respect to what stands in need of explanation. The traditional concern for thing-centred ontology is change. I do not expect an explanation of why my desk is very much as I left it when I was last in my office. For a process, on the other hand, persistence requires explanation. Physiology is largely concerned with understanding the multitude of internal processes that enable an organism to stay alive, to maintain its thermodynamic disequilibrium with its environment.

A clarification is needed at this point. When I refer to a process I shall, henceforth unless otherwise stated or obvious, mean an *individual* process, a process with the sort of coherence and persistence that might suggest treating it as a thing. Organisms, on my view, are paradigms of such coherent individual processes, though less controversially processual entities such as storms or rivers also have good claims to be individuals. Some processes—erosion, inflation, evolution—lack any such coherence. I shall not address philosophical doubts as to whether there even are individual processes that persist through time, though the discussion may give some indication of why such doubts arise and also of why they are misplaced.

The second reason why the processual status of organisms is important is that it places in the proper perspective the search for mechanistic explanation that is often alleged to be central to the contemporary life sciences [7,8]. I take a mechanistic explanation to be, very roughly, one that involves identifying a set of constituents of a phenomenon and showing how their actions and interactions combine to generate the phenomenon. There is no doubt that this has been an enormously productive scientific strategy. Nonetheless, from a process perspective the mechanisms postulated by such explanations must always be abstractions from the wider biological context, and this always poses potential limits on their application. First, the constituents of a biological mechanism are themselves dynamic and more or less transient entities. Mechanistic explanations will be successful only to the extent that the constituents identified are sufficiently stable on the timescale of the phenomenon under investigation. And second, biological processes are typically stabilized not just by the interactions of their parts, but also by interactions of the whole with its wider biological and abiotic context. These limitations do not imply that mechanistic explanations cannot be extremely illuminating; they do show that their success should not be taken as a sufficient reason for inferring that the organism really is an interlocking system of mechanisms. It is not.

I should note that contemporary mechanicists, or ‘new mechanists’ as they are widely known, are a diverse group with views that diverge in many ways from the very rough summary just offered. Machamer, Darden and Carver [7] acknowledge the ontological importance of processes, but as part of a dualistic ontology very different from that advocated here. Craver & Bechtel [9] explicitly address the question of interlevel explanation, though denying that there is anything properly described as downward causation. Recent work by Bechtel qualifies this sceptical view on downward causation (e.g. [10]) and generally endorses many of the positions here associated with process ontology. Bechtel's status as a new mechanist, however, is a matter of debate (W Bechtel 2017, personal communication). Since this is not, at any rate, a paper about mechanicism, I shall not attempt to explore these divergences and subtleties further.

The organism should not be seen as a hierarchy of interconnected things, but rather as a hierarchy of processes at molecular, cellular, tissue, organ, etc., levels, operating at different interlocking timescales [11]. At each level the more or less stable entities—molecules, cells, organs—are stabilized both by their internal activities and by their interactions with their wider environments. The organism itself, of course, is not the terminus of this hierarchy, but just one further component. The stability of the organism also depends in part on its dynamic relation to its biotic and abiotic environment.

## 3. What evolves?

Organisms do not, of course, evolve. Evolution relates to the distribution of the properties of organisms over time. What organisms? It is commonly said that the relevant group of organisms should constitute a lineage, and sometimes that the relevant lineage is a species, which can even be made true by fiat as in G. G. Simpson's [12] definition: ‘a lineage (an ancestral descendent sequence of populations) evolving separately from others and with its own unitary evolutionary role’. Technically, it is better to talk of populations, as a species may consist of a number of isolated populations, hence evolving separately, but for present purposes it will do no harm to speak of species. A reason for doing so is that it will be useful to connect with the extensive philosophical literature on the nature of species, reminding ourselves thereby that it is a matter of great uncertainty what constitutes the appropriate kind of coherent lineage. It is popularly supposed, reflecting the lasting influence of Ernst Mayr, that species are interbreeding groups of organisms. But we need only note that the vast majority of species, and all species for the first 80% or so of the history of life, are asexual to see that this account is seriously limited. (Perhaps Mayr's rather dismissive attitude to microbes has helped to direct attention from this embarrassment to his so-called Biological Species Concept.)

A rather different issue has been widely debated by philosophers of biology, namely the question whether species are kinds or, rather, individuals. Philosophers have traditionally taken species terms as paradigmatic classificatory terms, and hence as referring to all the things that satisfy the conditions of membership of the relevant kind. But Michael Ghiselin [13] and David Hull [14] have persuaded the majority of the philosophical community that species are, on the contrary, individuals. Species, according to Ghiselin and Hull, and in accordance with the influential cladistic school of systematics, are properly understood as branches of the phylogenetic tree.

I believe the species as individuals view is partly correct, though with two very important provisos. First, a branch of the phylogenetic tree is a process not a thing. Apart from subsuming the obvious point that any part of the phylogenetic tree is temporally extended and constantly changing, recognition of its processual character immediately addresses some serious concerns that have been raised about the species as individuals thesis. An obvious such objection is that the alleged parts of a species are highly discontinuous. How are they identified as parts? Ruse [15], a prominent critic of the species as individuals thesis, notes that the important point might be integration rather than actual physical connection between the parts of an individual, but then complains that where the only connection between the parts of a supposed individual species is descent, descent begins to look suspiciously like an essential property that serves to define a class. Indeed exactly this view was subsequently defended by Griffiths [16] and others.

For a species-as-*individual process* view, however, there is no problem to address. A process is necessarily extended in time, and causal relations between temporal stages, or between spatial parts of temporal stages, are required to provide it with whatever integrity it has. Descent is just such a causal connection. A similar problem arises with regard to ambiguity of boundaries. Species have somewhat vague boundaries both synchronically (hybridization,) and temporally (speciation). Again, while this is difficult to align with standard metaphysical accounts of an individual, it is no problem at all for a process. No one expects a thunderstorm or a battle to have precisely delineated boundaries.

In fact, similar problems apply to organisms. Anyone who believes in superorganisms, for example ant colonies, that may include, as well as various castes of ant, domesticated fungi and several essential consortia of microbes, is happy with discontinuous organisms. And the spectrum of degrees of integration with symbionts, from mitochondria, widely thought of as parts of their hosts, through genomically-reduced obligate symbionts such as *Wolbachia* and *Buchnera* and obligate but horizontally acquired symbionts to, finally, purely ecological mutualisms, makes it difficult to define unambiguous boundaries to the organism. Processes are more or less well integrated, more or less clearly demarcated. As Hull notes, ‘Most organisms do exhibit more internal organization than most species, but this difference is one of degree, not kind. Most species do not exhibit the internal organization common in vertebrate organisms, but the same can be said for plants as organisms. Most plants do not exhibit the internal organization common in vertebrate organisms’. [17, p. 32].

My second proviso perhaps deviates more strongly from the spirit of the species as individuals thesis. It is that while it is sometimes useful and correct to treat species as individuals, they can also, equally correctly be treated as classificatory terms. In fact, as I shall argue, it may very well be that some species can *only* be treated in the second way. The point here is that classification is a vital part of any scientific project, and especially vital in a domain with the vast diversity of biology. As I have argued elsewhere in more detail [18,19], the importance of classification provides special desiderata for distinguishing species, and these should not be outweighed by sometimes transient theoretical considerations. In short, species can be units of evolution, units within which evolutionary change takes place and as such should be seen as individual processes; but this cannot supplant their equal importance as units of classification (see also [20]).

## 4. Stabilization of species processes

If (some) species are individual processes, we should ask, as discussed above, what it is that maintains their coherence or integration over time. Note here that while not all processes need have either integration or individual status, to have the latter one must have the former. Geological erosion, for example, is a process with no integration; there is no temptation to divide it into distinct individuals. But if Hull and Ghiselin are right, species must be stabilized processes.

A first, and very important part of the answer to what makes species stable, is natural selection. It has often been proposed that most selection is stabilizing selection, and the continued production over sometimes very long periods of time of very similar phenotypes is generally attributed not to the perfection of the reproductive process, but to the greater selective success of a particular phenotype. As Reiss [21] persuasively argues, much of the importance of natural selection is most illuminatingly understood under the rubric of the *conditions of existence*, a phrase used by Darwin, but more often associated with Georges Cuvier. It is no trivial matter for an organism to satisfy the conditions of existence, and if the areas of morphospace that make this possible are very limited, natural selection will maintain homogeneous species. Darwin also famously observed the production of organisms far beyond the numbers required to maintain a species. Though this is generally remarked as part of the story of adaptive evolutionary change, it is also important that the stability of the species requires overproduction to compensate for the production of inviable individuals and the random losses of pre-reproductive individuals. The latter, in many cases, will constitute the overwhelming proportion of cases. Overproduction, in short, is necessary not just for adaptive evolutionary change, but also for stable maintenance of the lineage.

Natural selection is not, of course, sufficient to stabilize a species over time. Just as an organism must constantly renew the cells of which it is composed, so a species, qua individual, must replace the organisms that are its parts. The Modern Synthesis has understood this process of reproduction as, at its core, replication, and this is a central point of criticism for advocates of an extended, or more radically replaced, understanding of evolution. By replication here I mean exact copying, as is generally understood to occur when a DNA sequence serves as a template for an identical sequence. (For discussion of the distinction between reproduction and replication, see [22].) The quasi-digital nature of this process grounds the claim that this is *exact* copying, and underlies Richard Dawkins' rather strange claim that genes are immortal [23, ch. 3]: the nucleotide sequence can, in principle, be precisely replicated in perpetuity. With more or less hedging, the Modern Synthesis has taken this to be the overwhelmingly important part of reproduction, more or less explicitly, thereby, assuming that the DNA sequence was sufficient to determine the phenotype.

There is much more to reproduction, however, than replication. Reproduction means, as the etymology suggests, producing again, and there is no reason in principle why the production of a new organism in a lineage should involve the replication of anything. As a matter of fact it appears that terrestrial reproduction always involves nucleic acid sequence replication but, as various contributors to this volume have demonstrated (e.g. Muller on development; Stotz on parental effects; Jablonka on non-genetic inheritance), it involves much else besides. Moreover as Noble [6] emphasizes, the nucleic acid sequences that are generally thought of as targets of replication are only maintained in a persistent state by elaborate editing and correcting processes in the cell, and thus may themselves be better described as being reproduced.

The stability of a lineage, finally, depends crucially on its relations with the external environment. But rather than this being, as has often been supposed, something achieved by the passive adaptation of the evolving lineage to the demands of the environment, the organisms in a typical lineage do a great deal to adapt the environment to their needs, so-called niche construction [24,25]. This may amount to full-scale engineering of the environment [26], as in the classic examples of beaver dam building or coral reef formation, but may also take more local forms, such as nest building and burrow digging. In fact all organisms have some effect on their environment, and therefore on the conditions of existence that they must satisfy.

Niche construction is often compared to Richard Dawkins' [27] concept of the extended phenotype. For Dawkins the beaver's dam or bird's nest is part of the (extended) phenotype of the beaver or bird, encoded in its genes and expressed as the animal creates the external structure. Niche construction theorists, however, emphasize the bi-directionality of the relation. The altered niche affects the behaviour and ultimately drives the evolution of the organism.

The difference in these perspectives nicely illustrates the difference between a thing- and a process-centred ontology. The extended phenotype concept extends the boundaries of the object (organism), but these boundaries are still fully determined by that object's internal, intrinsic properties, and the lineage is just the sum of these objects. Seeing the organism, or in this case the lineage, as a process, on the other hand, we should expect its limits to be maintained by activities at its boundaries, as a living membrane actively transports numerous molecules to maintain the chemical discontinuity it marks, or the surrounding flows maintain a whirlpool. This is just the difference the niche construction perspective signals from the extended phenotype.

If species are processes of this kind, then evolution is the change within such processes. Stabilization of a process is always limited, so some such change is to be expected, as has been extensively discussed in accounts of drift. Where does adaptive change come from? A trivial but sometimes obfuscated point is that it never comes from natural selection. Selection cannot occur unless some other process provides alternatives to select from. It follows that any thesis about the power of natural selection to generate change implicitly presupposes a thesis about a process or processes that generate selectable change. A distinctive thesis in the Modern Synthesis is that the overwhelmingly predominant source of selectable change is small random mutations, and consequently views about the power of natural selection have sometimes smuggled in assumptions about the ability of cumulative small mutations to generate almost arbitrary degrees of phenotypic change. Contributors to this special issue describe various other sources of variation, and indeed of adaptive variation, so questions about the efficacy of particular sources including random mutation should be seen as open. I shall turn very briefly to enumeration of some sources of adaptive variation towards the end of this paper.

## 5. Kinds of lineage and degrees of integration

More or less stable, coherent lineages are not necessary for evolutionary change. The first 2.5 billion years of solely unicellular life were apparently characterized by asexual reproduction and promiscuous lateral transfer of genes between sometimes distantly related individuals. It is hard to see why there should be any well-distinguished, species-like sub-processes within this evolving whole. To the extent that there are strong divisions between kinds, this is likely to be because natural selection favours and disfavours particular areas of morphospace. Put differently, the combinations of traits that satisfy the conditions of existence occupy discontinuous regions of trait space. (This does rather oversimplify the matter, as the conditions of existence depend on what other organisms concurrently exist. But this should not significantly affect the main point.)

Sexual reproduction introduces something quite new, internal integration of the lineage. Sex involves both horizontal and vertical connections between members of a species: horizontal between sexual partners and vertical between parents and offspring. Boundaries between species reflect not merely the contingencies of adaptation, but the fact that species have more or less effective means of policing their boundaries. The importance of this policing was particularly stressed by Paterson's [28] mate recognition species concept, defining species in terms of the ways that members were distinguished from non-members for reproductive purposes. Surely this overestimates the effectiveness of this boundary-preserving activity and underestimates the frequency of hybridization and, for that matter, its important role in speciation [29,30]. But as already noted, vague boundaries are no problem or surprise between processes.

I suggest that the invention or emergence of sex is also the emergence of species as individuals. Without sex there are no horizontal relations between the members of a species and they are connected only by their ancestry. But unless every individual, or at least every individual with a minimal novelty (e.g. a point mutation), is the ancestor of a new species there must be some horizontal connections that establish a group of individuals as an appropriate set of ancestors to found a species, and we appear to be launched on an infinite regress. If there existed species-like processes prior to sexual reproduction, these lacked any coherence or integration that could qualify them as processual individuals with persistence as such through time. This proposal also puts Mayr's familiar biological species concept in a slightly different light. Reproductive connections are indeed fundamental to the existence of species *as individuals*.

Sex is a minimal condition for a species to form as a coherent individual. In many, perhaps most, sexual species it provides all the coherence that there is. This is generally the case, at any rate, for those species that ecologists have described as r-selected, species, that is to say, that produce very large numbers of offspring of which a tiny fraction will survive. (The distinction between r- and K-selected species has been largely abandoned by ecologists, in recognition of the fact that there is a continuum of intermediate cases. Here I use the terminology only to indicate the extremes of this spectrum.) In such species there is minimal parental investment in offspring, and little opportunity for the emergence of culture or sociality. Frequently the contact between sexual partners is also minimal, sometimes in great danger of slipping into the relation of predator and prey. (I shall return shortly to those great niche constructors, the social insects.)

It is true that fairly r-selected species may well affect their niches, and may do so in ways that are advantageous to themselves. An excellent example are the earthworms studied in great detail by Charles Darwin [31]. The typical earthworm is, in many ways, more adapted to an aquatic than to a terrestrial life. But by its manipulation of the soil, notably the constant introduction of decaying organic matter, it keeps the soil wet enough to meet its adaptive requirements. It is unclear whether this is properly seen as a species-maintaining activity. There are many species of earthworm, so there is no species-specific benefit to their alterations of the environment. It is an interesting speculation that such processes of niche construction can create partially coherent supra-specific lineages at a much higher level than the reproductively connected lineage. But I shall not pursue that thought here. It seems likely that the kind of local and focused niche construction exemplified by beavers or nest-building birds is not found except where there is major parental investment in offspring, though I certainly do not rule out the possibility that more broadly directed kinds of niche construction may make important contributions to species coherence.

With K-selection, the strategy of producing much smaller numbers of offspring and investing heavily in their development, new forms of integration become possible. While some extragenetic maternal effects, mediated by molecules transferred to the oocyte, are possible even for strongly r-selected species, substantial periods of child-rearing allow far greater possibilities for parental, most commonly maternal, influence on the developing phenotype. The widely recognized phenomenon of phenotypic plasticity [32] provides ample opportunities for the mother to divert the offspring's development into directions that are adaptive in the context of perceived environmental conditions. Wolf and Wade [33] define maternal effects as a causal connection between some aspect of the mother's genotype or phenotype, and the phenotype of the offspring. Clearly the extended period of parental care in many vertebrate species provides many opportunities for such causal connections, and processes that allow parents to direct development in adaptive directions will be strongly selected. It seems likely *a priori* that such opportunities would be exploited, and the evidence supports this expectation [34].

One such process is epigenetic modification of the offspring's genome. Some kind of epigenetic system seems inevitable for a multicellular organism with highly differentiated cell lineages. The existence of such a system, in turn, provides a set of levers by which the parent (or any other aspect of the developmental environment) can influence the developmental trajectory of the organism. It again seems *a priori* plausible that parents would come to exploit these levers in adjusting the development of their young to changeable environmental conditions. And again this appears to have happened. A classic instance is the study of maternal care and its effect on the behavioural dispositions of rat pups by Meaney and colleagues [35,36].

Parental care provides opportunities for highly targeted niche construction, targeted, specifically, on the immediate environment of the offspring. Birds' nests provide a paradigm of this sort of activity, but social insect colonies remind us that this kind of niche construction is not necessarily tied to the kind of intergenerational relations found in vertebrates. This is becoming a familiar aspect of current evolutionary thinking [24,25] though the profound significance of replacing a picture in which the evolving lineage reacts passively to the environment, with one in which the lineage simultaneously shapes the environment to which it adapts, is not always sufficiently appreciated.

Parental care also provides unparalleled opportunities for enculturation, and hence for the evolution more generally of culturally transmitted behaviour. Such behaviour may also have physiological effects, for example mediated by epigenetic modifications. There is no reason in principle why a strongly r-selected species might not develop some kind of culture, and for all I know there may be examples of this. Nonetheless, it seems unlikely that there could be any very complex culture in the absence of the systematic collocation provided by parental care. Culture, in any case, provides a new channel for both horizontal and vertical transmission and evolution of behavioural traits.

A further crucial feature that adds a new dimension of integration to many K-selected lineages is sociality, the development of various more or less cooperative relations between individuals beyond parents and offspring. Though this is a complex and controversial subject, the existence of sociality is an indisputable empirical fact. It is widely though not universally believed that sociality creates supra-organismic level entities that can be selected [37].

In most social species it is assumed that social groups are disjoint: every individual is a member of at most one social group; and it could be argued that in that case sociality does not add to the integration of the species, but only adds an intervening level of organization between the organism and the species. This is patently not the case, however, for humans. Typical humans are involved in numerous social groups, more or less cooperative and more or less significant to the course of their lives. Humans belong simultaneously to families, organizations, companies, clubs, churches, political parties, etc., and thus the species is connected by a mass of criss-crossing and overlapping links and supra-organismic level entities.

This kind of social integration may be unique to humans, indeed even to modern humans in complex civilizations. It is perhaps part of the reason why some (e.g. [38]) have thought that humans exemplify the very special kind of sociality known as *eusociality*. The paradigms for eusociality are the social insects, numerous species of Hymenoptera (ants, bees and wasps) and Isoptera (termites). It is also said to be found in two mammalian species (of mole rats) a few other insect species, and a few crustaceans. The most distinctive feature of eusociality is the division of reproductive from non-reproductive labour, with specialist reproducers and communal care of the young by non-reproducers. There is often much further division of labour into so-called castes. Such systems provide a highly effective context for shaping the development of the young in various behaviourally modulated ways. While humans certainly do not have a distinct reproductive caste, they do have a more elaborate division of labour by far than any other species. So although eusocial species have the most clear cut supra-organismic level of organization, it is equally clearly a disjoint division into social wholes. Humans may be unique in having a species-wide network of cooperative and group-forming relations, and may therefore reasonably be claimed to be the most fully integrated species we know.

A central aspect of the move from a mechanistic thing ontology to a process ontology is that the commitment to strictly bottom up causal influences, from parts to wholes, is replaced with a recognition that whole systems can contribute to determining the properties of their parts. It is, therefore, likely that the emergence of the species as an integrated individual will affect the behaviour of organisms, its parts. The most obvious relevant examples come from niche construction, and the most obvious specific case is that of *Homo sapiens*. Modern humans live in a constructed niche that is necessary for a large proportion of the behaviour they undertake, and acquire the capacities they have in a constructed developmental niche including hospitals, schools, and a great deal else besides. It is not, of course, the species as a whole that produces these resources, but they are made possible by numerous distributed parts of the species, generating a remarkable degree of effective cooperation.

In sum, although any lineage may be said to be a process of a sort, the degree of integration of these processes is very varied. And hence the degree to which these processes may count as persistent individuals, or continuants, is very varied. Pace Hull and Ghiselin, not all species are individuals. It seems plausible that the kind of process that constitutes a particular lineage may have important implications for the evolutionary processes that it is liable to undergo.

## 6. Implications

Evolutionary change requires sources of novelty. Although the debate over the current status of the Modern Synthesis is often presented as a debate about the importance of natural selection, this is misleading. As I have noted, natural selection cannot create anything. When theorists applaud the power of natural selection, what they are really doing is remarking on the poverty of the sources of change with which selection has to work, these being restricted to small random changes in the genome. In the debate over the adequacy of the Modern Synthesis, questions arise whether certain kinds of change happen or not, notably changes with some inherent tendency to be adaptive (Lamarckianism); and also whether kinds of changes that are acknowledged to happen are available to evolution by natural selection. The latter question tends to revolve around the adequacy of the modes of inheritance that are supposed to embed the relevant changes in a lineage.

Numerous sources of evolutionary novelty have been proposed (here I do not mean by ‘novelty’ any particular exceptional *degree* of novelty). The Modern Synthesis typically restricts these to genetic changes, notably mutation and recombination, but in principle also lateral acquisition of genetic material, though often this last is argued to be of relatively small importance. (Even very occasional lateral acquisition could be disproportionately important, however, as it might come, as is familiar in bacteria, with pre-packaged functionality. The vast numbers of viruses and similar entities in the biosphere provide a plausible means for such acquisition.)

In the microbial world, where the processes I have been discussing that account for the emergence of species, or lineages, as individuals do not occur, it is plausible that pretty much the standard Modern Synthesis model of genetic change and selection is sufficient to account for evolutionary change. As microbial evolution is all there was for 80% of the history of life, this is no minor concession. It is again important, however, to note the potential significance of lateral acquisition of genetic material. Microbes evolved in a context in which a far wider pool of genetic resources was potentially available than merely those in their own lineage, narrowly conceived. On the other hand, the price paid for this, one might say, was the impossibility of establishing higher-level entities, integrated lineages.

The emergence of sex in eukaryotes, at least 1.2 billion years ago [39], made possible the appearance of species as persisting individuals. Rescher [2] remarks, ‘For process philosophy, what a thing is consists in what it does’, so if sexually integrated species are indeed individual processes, we might wonder whether there is anything they do, beyond just persisting through time. The answer to this question might even offer a fresh perspective on the long debated question of why sex evolved at all.

The immediate answer to the question what species (or strictly, as noted earlier, populations) do is, of course, evolve. But the capacity to evolve preceded the appearance of sex, so what we should consider is whether the species as individual provides enhanced evolvability. Moreover, since sexual reproduction provides a boundary to the species, and a barrier to the acquisition of external genetic material, it appears prima facie to reduce evolvability. So if evolvability is indeed an advantage that partly explains the persistence and increasing dominance of sexual species, we might expect the gains in this regard to be substantial. The ability of advantageous genetic features to spread more rapidly through a species, and the ability, through recombination, of several advantageous alleles to be selected simultaneously, are sometimes proposed as decisive advantages of sex. However, this does little to explain the evolution of K-selected sexual species, where such advantages seem only a minor compensation for the great losses in this respect due to slow reproductive processes and small numbers of offspring.

If the highly integrated species is indeed a vehicle for greater evolvability, it is surely because it provides new sources of selectable variability. And indeed there are many familiar phenomena, already discussed above and in other essays in this volume, that offer to provide just this.

First, integrated species appear to offer a much more favourable environment for the transition from intra-specific competition to cooperation, as exemplified in the very high levels of cooperation found in eusocial species and in humans. In the former case, especially in the eusocial insects, it is widely accepted that the integrated colonies are a kind of organism (or ‘superorganism’) and clearly they have capacities far beyond those of their constituent individuals. The striking success of these insects and indeed of humans testifies to the evolutionary success of this kind of cooperation.

The extended care found in K-selected species provides an opportunity for a developmental system with multiple inputs in addition to the material of reproduction [40,41]. These include the environmental inputs made possible by the niche constructing activities of previous and present conspecifics and a wide variety of parental effects. They also provide an opportunity for the transmission of sometimes complex cultural traditions. All of these aspects of the developmental system are in principle entirely heritable, and thus provide potential pathways of evolutionary change. Niche construction and maintenance activities, or parenting activities can be learned and passed down the generations, and culture may be passed down through this and other routes in a more widely social species. This evolution may be solely behavioural, but it may also be physiological through the epigenetic direction of developmental plasticity.

It is hard to deny, though there is a very powerful ideological tendency to do so, that much evolutionary change through these pathways has the potential to be both acquired and adaptive. At the most uncontroversial end is human culture. We can argue, of course, whether it is a good thing, but that innovations in food production, say, are introduced because they produce more food, is uncontroversial. Much behavioural innovation that has been observed in other primates—food washing, termite fishing, and so on—has a similar character. How widespread this is is not something I shall discuss here. The point is only that a more integrated species does indeed provide multiple new evolutionary pathways that have in demonstrable instances resulted in adaptive evolutionary change.

There is a curious tendency to dismiss all such evolutionary pathways on the grounds that they are too transient and allegedly less durable than genetic change. Perhaps this tendency has been encouraged by Dawkins's already remarked appeal to immortality [23, ch. 3] in his argument for the overwhelming evolutionary importance of DNA. It is at any rate extraordinary that one should require the explanation of a changing process to be grounded in unchanging causes, and perhaps can be seen as a paradigm of the misleading effects of a substance- rather than properly process-based ontology.

One further key point is the following. Species are a diverse category. Arguably they are ontologically diverse, encompassing both processes and kinds, as profound a diversity as imaginable. More prosaically, even as concrete entities, they differ in very significant respects. If species are what evolve, we should not, for this reason, expect quite general accounts of evolution. The Modern Synthesis, specifically, may be more or less true for some kinds of species, but quite inadequate for others. If species have evolved new forms of evolvability, this is surely to be expected. Evolvability of many populations may just be a summative property of organism properties, but as species become integrated processes it is plausible that evolvability might emerge as a specific capacity of lineages.

This leads me to a more speculative final thought. There is a philosophical tradition of seeing organisms as a kind of agent, as beings in some way autonomously pursuing their own goals or interests. Denis Walsh [42] argues that this is a vital part of an organism-centred view of evolution of the kind championed by Darwin, and as opposed to contemporary molecule-centred views. Substance- (or thing-) based thinking has struggled with the idea of organisms as agents, and has often considered that at most humans achieved this rarefied status. For a process, intrinsically dynamic, and dynamic in ways that conduce to the persistence of the process, agency is a much more natural attribution. Hence process thinkers, such as the mid-twentieth century organicists [43–45] thought agency a quite general feature of organisms. If some species are themselves living processes, might they themselves have a kind of agency, inherent tendencies to change (act) in ways that promote their survival? If we take seriously the claim that species are individuals then this is at least a possibility worth investigation.

## Data accessibility

This article has no additional data.

## References

[1] Woodger JH 1929 Biological principles. London, UK: K. Paul, Trench, Trubner.

[2] Rescher N 2004 Process philosophy, *The Stanford encyclopedia of philosophy* (Spring 2004 Edition) (ed. Edward N Zalta), http://plato.stanford.edu/archives/spr2004/entries/process-philosophy/

[3] Dupré J 2012 Processes of life: essays in the philosophy of biology. Oxford, UK: Oxford University Press.

[4] Robinson H 2014 Substance, *The Stanford encyclopedia of philosophy* (Spring 2014 Edition) (ed. Edward N Zalta), https://plato.stanford.edu/archives/spr2014/entries/substance/

[5] Dupré J 2010 The polygenomic organism. In Nature after the genome (eds Parry S, Dupré J), pp. 19–31. Malden, MA: Blackwell Publishing, Sociological Review Monograph series.

[6] Noble D 2017 Evolution viewed from physics, physiology and medicine. Interface Focus 7, 20160159 (10.1098/rsfs.2016.0159) 28839924 PMC5566812

[7] Machamer PK, Darden L, Craver CF 2000 Thinking about mechanisms. Philos. Sci. 67, 1–25. (10.1086/392759)

[8] Craver CF, Darden L 2013 In search of mechanisms: discoveries across the life sciences. Chicago, IL: University of Chicago Press.

[9] Craver CF, Bechtel W 2007 Top-down causation without top-down causes. Biol. Philos. 22, 547–563. (10.1007/s10539-006-9028-8)

[10] Bechtel W 2017 Explicating top-down causation using networks and dynamics. Philos. Sci. 84, 253–274. (10.1086/690718)

[11] DiFrisco J 2016 Time scales and levels of organization. Erkenntnis. (10.1007/s10670-016-9844-4)

[12] Simpson GG 1961 Principles of animal taxonomy. New York, NY: Columbia University Press. 10.1126/science.133.3464.1589 17781120

[13] Ghiselin M 1974 A radical solution to the species problem. Syst. Zool. 23, 536–544. (10.2307/2412471)

[14] Hull D 1978 A matter of individuality. Philos. Sci. 45, 335–360. (10.1086/288811)

[15] Ruse M 1987 Biological species: natural kinds, individuals, or what? Br. J. Philos. Sci. 38, 225–242. (10.1093/bjps/38.2.225)

[16] Griffiths PE 1999 Squaring the circle: natural kinds with historical essences. In Species: new interdisciplinary studies (ed. Wilson RA). Cambridge, MA: MIT Press.

[17] Hull D 1999 On the plurality of species: questioning the party line. In Species: new interdisciplinary essays (ed. Wilson RA), pp. 3–20. Cambridge, MA: MIT Press.

[18] Dupré J 1994 The philosophical basis of biological classification. Review of Marc Ereshefsky (ed.), The units of evolution: essays on the nature of species. Stud. Hist. Philos. Sci. 25, 271–279. (10.1016/0039-3681(94)90032-9)

[19] Dupré J 2001 In defence of classification. Stud. Hist. Philos. Biol. Biomed. Sci. 32, 203–219. (10.1016/S1369-8486(01)00003-6)

[20] Reydon T 2003 Species are individuals or are they? Philos. Sci. 70, 49–56. (10.1086/367868)

[21] Reiss J 2009 Not by design: retiring Darwin’s watchmaker. Berkeley, CA: University of California Press.

[22] Wilkins JS, Hull D 2014 Replication and reproduction, *The Stanford encyclopedia of philosophy* (Spring 2014 Edition), (ed. Edward N Zalta), http://plato.stanford.edu/archives/spr2014/entries/replication/.

[23] Dawkins R 1976 The selfish gene. Oxford, UK: Oxford University Press.

[24] Laland K, Odling-Smee J, Endler J 2017 Niche construction, sources of selection and trait coevolution. Interface Focus 7, 20160147 (10.1098/rsfs.2016.0147) 28839920 PMC5566808

[25] Odling-Smee FJ, Laland KN, Feldman MW 2003 Niche construction: the neglected process in evolution. Princeton, NJ: Princeton University Press.

[26] Jones CG, Lawton JH, Shachak M 1994 Organisms as ecosystem engineers. Oikos 69, 373–386. (10.2307/3545850)

[27] Dawkins R 1982 The extended phenotype. Oxford, UK: Oxford University Press.

[28] Paterson HEH 1985 The recognition concept of species. In Species and speciation, vol. 4 (ed. Vrba ES), pp. 21–29. *Transvaal Mus. Monogr* Pretoria, South Africa: Transvaal Museum.

[29] Pennisi E 2016 Shaking up the tree of life. Science 354, 817–821. (10.1126/science.354.6314.817) 27856860

[30] Mallet J 2008 Hybridization, ecological races and the nature of species: empirical evidence for the ease of speciation. Phil. Trans. R. Soc. B 363, 2971–2986. (10.1098/rstb.2008.0081) 18579473 PMC2607318

[31] Darwin C 1881 The formation of vegetable mould, through the action of worms, with observations on their habits. New York, NY: D. Appleton.

[32] West Eberhard MJ 2003 Developmental plasticity and evolution. New York, NY: Oxford University Press.

[33] Wolf JB, Wade MJ 2009 What are maternal effects (and what are they not)? Phil. Trans. R. Soc. B 364, 1107–1115. (10.1098/rstb.2008.0238) 19324615 PMC2666680

[34] Stotz K 2017 Why developmental niche construction is not selective niche construction and why it matters. Interface Focus 7, 20160157 (10.1098/rsfs.2016.0157) 28839923 PMC5566811

[35] Meaney MJ, Szyf M, Seckl JR 2007 Epigenetic mechanisms of perinatal programming of hypothalamic–pituitary–adrenal function and health. Trends Mol. Med. 13, 269–277. (10.1016/j.molmed.2007.05.003) 17544850

[36] Champagne FA, Meaney MJ 2006 Stress during gestation alters postpartum maternal care and the development of the offspring in a rodent model. Biol. Psychiatry 59, 1227–1235. (10.1016/j.biopsych.2005.10.016) 16457784

[37] Sober E, Wilson DS 1999 Unto others: the evolution and psychology of unselfish behavior. Cambridge, MA: Harvard University Press.

[38] Wilson EO 2012 The social conquest of earth. New York, NY: Liveright Publishing.

[39] Butterfield NJ 2000 *Bangiomorpha pubescens* n. gen., n. sp.: implications for the evolution of sex, multicellularity, and the Mesoproterozoic/Neoproterozoic radiation of eukaryotes. Paleobiology 26, 386–404. (10.1666/0094-8373(2000)026%3C0386:BPNGNS%3E2.0.CO;2)

[40] Oyama S 1985 The ontogeny of information: developmental systems and evolution. Durham, NC: Duke University Press.

[41] Oyama S, Griffiths PE, Gray RD 2001 Cycles of contingency: developmental systems and evolution. Cambridge, MA: MIT Press.

[42] Walsh D 2015 Organisms, agency and evolution. Cambridge, UK: Cambridge University Press.

[43] Russell ES 1924 The study of living things: prolegomena to a functional biology. London, UK: Methuen.

[44] Haldane JS 1931 The philosophical basis of biology. London, UK: Hodder & Stoughton.

[45] Bertalanffy L 1952 Problems of life: an evaluation of modern biological and scientific thought. New York, NY: Harper & Brothers.

